# Differential protein expression in human corneal endothelial cells cultured from young and older donors

**Published:** 2008-09-30

**Authors:** Cheng Zhu, Ian Rawe, Nancy C. Joyce

**Affiliations:** Schepens Eye Research Institute and Department of Ophthalmology, Harvard Medical School, Boston, MA

## Abstract

**Purpose:**

To establish a baseline protein fingerprint of cultured human corneal endothelial cells (HCEC), to determine whether the protein profiles exhibit age-related differences, and to identify proteins differentially expressed in HCEC cultured from young and older donors.

**Methods:**

Corneas were obtained from five young (<30 years old) and five older donors (>50 years old). HCEC were cultured, and protein was extracted from confluent passage 3 cells. Extracts from each age group were pooled to form two samples. Proteins were separated on two-dimensional (2-D) gels and stained with SyproRuby. Resultant images were compared to identify protein spots that were either similarly expressed or differentially expressed by at least twofold. Protein spots were then identified by matrix-assisted laser desorption/ionization time of flight (MALDI-TOF) mass spectrometry.

**Results:**

Protein spots were well resolved, and patterns were reproducible on 2-D gels using either pH 3–10 or pH 4–7 IPG strips. Two-dimensional gels prepared with pH 4–7 IPG strips were used for differential display analysis, which was reproduced on three separate pairs of gels. MALDI-TOF identified 58 proteins with similar expression; 30 proteins were expressed twofold higher in HCEC from young donors; five proteins were expressed twofold higher in cells from older donors; and 10 proteins were identified in gels from young donors that did not match in gels from older donors. Several proteins expressed at higher levels in younger donors support metabolic activity, protect against oxidative damage, or mediate protein folding or degradation.

**Conclusions:**

This is the first proteomic comparison of proteins expressed in HCEC cultured from young and older donors. Although restricted to proteins with isoelectric points between pH 4.0 and pH 7.0, the data obtained represent an initial step in the investigation of molecular mechanisms that underlie physiologically important age-related differences in cultured HCEC, including differences that may affect proliferative capacity. Results indicate that HCEC from older donors exhibit reduced expression of proteins that support important cellular functions such as metabolism, antioxidant protection, protein folding, and protein degradation. These differences may affect the ability to consistently obtain a sufficient number of healthy cultured HCEC for use in preparing bioengineered endothelium as an alternative method for the treatment of endothelial dysfunction.

## Introduction

Corneal endothelium is a physiologically important monolayer of cells that functions to maintain corneal transparency. Human corneal endothelial cells (HCEC) in vivo do not normally divide to replace cells lost as the result of disease [1,2] or trauma [3,4]. Instead, wound healing occurs by migration and/or enlargement of neighboring cells [5]. If cell loss exceeds a threshold limit, the integrity of the endothelium becomes compromised, resulting in painful corneal edema and loss of visual acuity. Although penetrating keratoplasty remains the treatment of choice to restore clear vision following the critical decline in endothelial cell number, there are several new treatment strategies currently being explored. These include modified posterior lamellar keratoplasty strategies such as Deep Lamellar Endothelial Keratoplasty (DLEK) [6], Descemet's stripping with endothelial keratoplasty (DSEK) [7], and Descemet Membrane Endothelial Keratoplasty (DMEK) [8]. These newer methods offer some advantages over full-thickness corneal transplantation but can suffer from inadequate donor tissue attachment, and endothelial cell loss can be accelerated within these transplants due to increased tissue manipulation during surgery [9-11].

Because the aging population requiring corneal transplants is increasing and the donor age requirements and tissue quality limit the availability of donor corneas, there is increased interest in alternative approaches to restore corneal transparency following loss of endothelial function. Several new approaches are taking advantage of the fact that, although HCEC do not divide in vivo, they retain proliferative capacity [12-14]. One approach is to directly stimulate proliferation either in vivo as a means of directly increasing endothelial cell density (ECD) or in ex vivo donor corneas to induce cell division in the endothelium of donor corneas with unacceptably low ECD [15]. This treatment would potentially increase the number of donor corneas available for transplantation. Tissue bioengineering is another promising approach in which HCEC are cultured on a suitable substrate to increase cell numbers and then transplanted to replace diseased or damaged endothelium [16,17]. Ideally, it would be best to use a patient’s own cells as a source for this bioengineered tissue. An alternative method is to culture HCEC from donor corneas to expand cell numbers for use in the bioengineered constructs. Because the majority of patients requiring treatment for endothelial dysfunction are older and the majority of available corneas are from older donors, it is important to obtain more information regarding the basic cell biology of corneal endothelium from young and older donors so that optimal methods to increase ECD can be developed for use in tissue bioengineering.

Previous studies from this laboratory have used an ex vivo corneal wound healing model to compare the relative proliferative capacity of HCEC from young (<30 years old) and older donors (>50 years old) [12]. Results indicate that HCEC from older donors can proliferate but do so more slowly than cells from younger donors. Studies of cultured HCEC have shown a similar age-related growth response in which the population doubling time for HCEC from young donors was 46.25 h (range: 27–59 h) and from older donors was 90.25 h (range: 81–101 h)—the difference being statistically significant (p=0.0016) [14,18]. In addition, the density of confluent primary cultures of HCEC from young donors is nearly three times higher than that of cultures from older donors [18]. The molecular basis for this age-related difference in proliferative capacity appears to involve an age-dependent increase in the expression of the cyclin-dependent kinase inhibitors, p21Cip1 and p16INK4a, which reduce the ability of mitogens to stimulate cell cycle progression [19]. Other age-related functional alterations have been reported for HCEC, including an increase in the permeability of HCEC to fluorescein [20] and a decrease in pump function [21].

Proteomics technology is being widely used in research fields such as aging [22], biomarker discovery [23,24], and new drug development [25]. Proteomics is also being applied in the field of eye research, including studies of the cornea [26-33]. In the current study, proteomic analysis was used to compare relative protein expression in confluent cultured HCEC from young and older donors. Goals were to establish a baseline protein fingerprint of cultured HCEC, to determine whether the protein profiles show age-related differences, and to identify proteins that are differentially expressed in HCEC cultured from young and older donors. The results of these studies should provide information that could lead to the development of methods to consistently obtain healthy, cultured HCEC, which can be used for the preparation of bioengineered tissue with consistently high cell density.

## Methods

### Materials

OptiMEM-I, minimum essential medium (MEM), Dulbecco’s phosphate-buffered saline (PBS), gentamicin, and trypsin/EDTA were purchased from Gibco BRL/Life Technologies (Rockville, MD). Bovine pituitary extract (also known as keratinocyte growth supplement) was from Biomedical Technologies (Stoughton, MA). Epidermal growth factor (EGF; from mouse submaxillary glands) was obtained from Upstate Biotechnologies (Lake Placid, NY). Fetal bovine serum (FBS) was from Hyclone (Logan, UT). Ascorbic acid, chondroitin sulfate, calcium chloride, 0.02% EDTA solution (EDTA disodium salt), and antibiotic/antimycotic solution were purchased from Sigma (St. Louis, MO). Sequential Extraction Reagent III (ER3), Protein Assay Dye Reagent, mineral oil, and ReadyPrep^TM^ rehydration/sample buffer were obtained from Bio-Rad (Hercules, CA). SyproRuby was purchased from Invitrogen (Carlsbad, CA).

### Culture of human corneal endothelial cells

Five pairs of human corneas from young donors (<30 years old) and five pairs from older donors (>50 years old) were obtained from the National Disease Research Interchange (NDRI, Philadelphia, PA) and formed two age comparison groups. Donor confidentiality was maintained by the original eye bank, NDRI, and by this laboratory according to the tenets of the Declaration of Helsinki. Donor information is presented in Table 1. Exclusion criteria were the same as previously published [18]. Corneas were accepted for study only if the donor history and condition of the corneas indicated no damage to the health of the endothelium. Endothelial cell counts for all accepted corneas were at least 2000 cells/mm^2^. Corneas were not accepted for study if the period between time of death and time of preservation was too long (>12 h) or if guttae or other endothelial abnormalities were noted. Corneas were not accepted from donors with glaucoma, sepsis, ocular infection, or on large doses of chemotherapeutic agents. Primary culture and subculture of HCEC followed previously described protocols [13,14]. Passage 3 HCEC from each donor were maintained in two T75 flasks for at least one week after reaching confluence to ensure cell-cell contact-dependent inhibition of proliferation.

**Table 1:** Donor information.

**Age**	h*	Days**	**Cause of Death**
13	3	2	Motor vehicle accident
14	5	6	Intracranial hemorrhage
16	2	3	Motor vehicle accident
20	10	2	Multiple trauma
20	5	7	Angelman's syndrome
55	12	2	Thrombocytopenia
67	2	7	Intracranial bleeding
69	2	2	Triple aneurysm
69	10	1	Myocardial infarction
70	12	1	Cerebrovascular accident

The asterisk indicates that the data in the column are the periods of time (h) passed between the deaths of the donors and preservation of their cornea. A double asterisk means that the data in the column are the number of days from preservation of the donor corneas to culture.

### Protein sample preparation

Confluent passage 3 cells were rinsed with PBS to remove residual culture medium. Cell scrapers were used to remove cells from the culture plates. Harvested cells were centrifuged at 5,000 rpm for 10 min to form firm pellets. Bio-Rad Sequential Extraction Reagent III (ER3) with 1% tributyl phosphine (reducing agent) was added, and the cells were gently pipetted up and down for 1–2 min followed by incubation at room temperature for about 10 min to ensure thorough protein solubilization. Soluble proteins were harvested after centrifugation at 40,000 rpm at room temperature for 1 h and then stored at −80 °C until further analysis.

### Two-dimensional gel electrophoresis

Before gel electrophoresis, equal amounts of protein from five donors per age group were pooled to form two final samples. Protein concentrations of the two, pooled samples were determined by a modified Bio-Rad protein assay. The extracted protein together with De-Streak reagent (GE Healthcare, London, UK) and bromophenol blue (Bio-Rad) was loaded onto pH 3–10 or pH 4–7 linear gradient IPG strips of 17 cm (Bio-Rad). Active rehydration was performed for at least 16 h following the Bio-Rad protocol before isoelectric focusing (IEF) in a Protean IEF Cell (Bio-Rad). IEF was run for a total of 60,000 Vhr after reaching maximum 10,000 voltages. The IPG strip was further incubated with equilibration buffers I and II (Bio-Rad) for 15 min each. Proteins were then separated on 8%–16% polyacrylamide pre-cast gels of 19 cm (Bio-Rad) using a Protean II apparatus (Bio-Rad). Electrophoresis was run until bromophenol blue dye just started to disappear from the bottom of the gel (approximately 1440 Vhrs). Gels were fixed for 1 h in 10% methanol and 7% acetic acid solution, stained overnight with SyproRuby protein gel stain, and washed for 1 h in water. Protein spots were then imaged with a ProXPRESS Proteomic Imaging System (Perkin Elmer, Boston, MA) using excitation (480/30) and emission (620/30) filters to visualize the SyproRuby.

### Gel image analysis

Images from gels prepared using pH 4–7 IPG strips were analyzed using ProFinder version 2005 software (Nonlinear Dynamics, Newcastle upon Tyne, UK). Automatic analysis wizard was used, and spot editing was performed according to the software instructions. After warping to align spots between gels, manual inspection and editing, and automatic background subtraction, the normalized volumes of individual protein spots were compared. For these analyses, the two-dimensional (2-D) gel containing proteins extracted from the HCEC of young donors was used as the reference gel. Density differences between spots were confirmed though the use of various tools such as “montage window” and three-dimensional (3-D) topographic mapping. The software then generated lists indicating protein spots in which the normalized volume was similar (within a twofold range), spots that showed at least a twofold difference in normalized volume between samples, and spots that only appeared in one of the two samples.

### Protein identification by MALDI-TOF-MS

Gel plugs containing the protein spots of interest were picked from the 2-D gels using a spot-picking robot equipped with a CCD camera (ProXCISION; Perkin Elmer) and filter sets for SyproRuby. Plugs were placed in a ZipPlate (Millipore, Billerica, MA), dehydrated with 100% acetonitrile for 15 min, rehydrated in 15 µl of 25 mM ammonium bicarbonate, which contained 100 ng Trypsin Gold (Promega, Madison, WI), and then incubated at 30 °C overnight. The C_18_ resin of the ZipPlate was then activated with 9 µl acetonitrile for 15 min at 37 °C. Peptides were then washed out of the gel plug with 180 µl 0.1% trifluoroacetic acid (TFA) for 30 min and bound to C_18_ resin using low vacuum followed by two washings with 100 µl TFA under high vacuum. Peptides were then directly eluted onto a disposable MALDI target plate (Perkin Elmer) by direct vacuum elution with matrix α-cyano-4-hydroxy cinnamic acid (α-CHCA at 10mg/ml; LaserBiolabs, Sophia-Antipolis Cedex, France) in 50% acetonitrile/50% TFA. The matrix was allowed to air-dry allowing crystals to form. Peptide mass fingerprints were obtained on a Perkin Elmer prOTOF MALDI mass spectrometer, and data was searched on a local copy of the NCBI protein database (National Center for Biotechnology Information) using the ProFounder search engine (Rockefeller University, New York, NY). Identified peptides were grouped into the smallest set of non-redundant proteins possible. Different forms (charge states and modifications) of the same peptide were compressed into a single hit. Protein identifications were reviewed manually to ascertain their accuracy and to ensure that consistent database entries were reported.

## Results

### Characteristics of cultured human corneal endothelial cells

**Figure 1:**
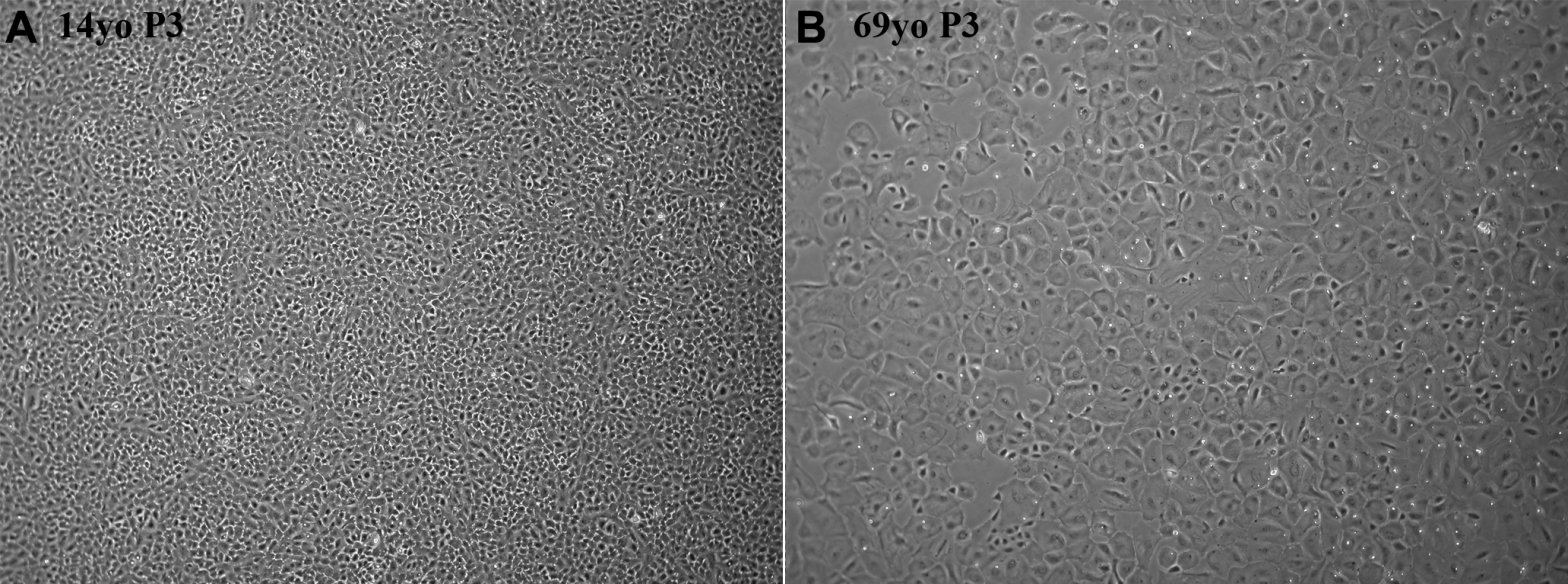
Representative phase-contrast images of confluent passage 3 human corneal endothelial cells cultured from a 14-year-old donor and a 69-year-old donor. HCEC from these donors were among those used for subsequent proteomic analysis. Cells cultured from older donors were consistently larger and displayed more variable shapes than cells from young donors. Original magnification: 4X.

Age-related differences in morphology and growth characteristics of HCEC observed in this study were very similar to those previously described [13,14,18]. Figure 1 presents representative phase-contrast images of confluent HCEC cultured from a young and an older donor demonstrating typical age-related differences in morphology. At confluence, cells cultured from the young donor formed a tightly-packed monolayer of generally polygonal cells whereas HCEC cultured from the older donor were much larger, is reflecting an overall reduction in cell density, and displayed a greater variability in cell shape. The growth rate of HCEC from older donors was consistently slower with the overall doubling time similar to that reported previously [18].

### Two-dimensional gel protein separation of extracts from cultured human corneal endothelial cells

**Figure 2:**
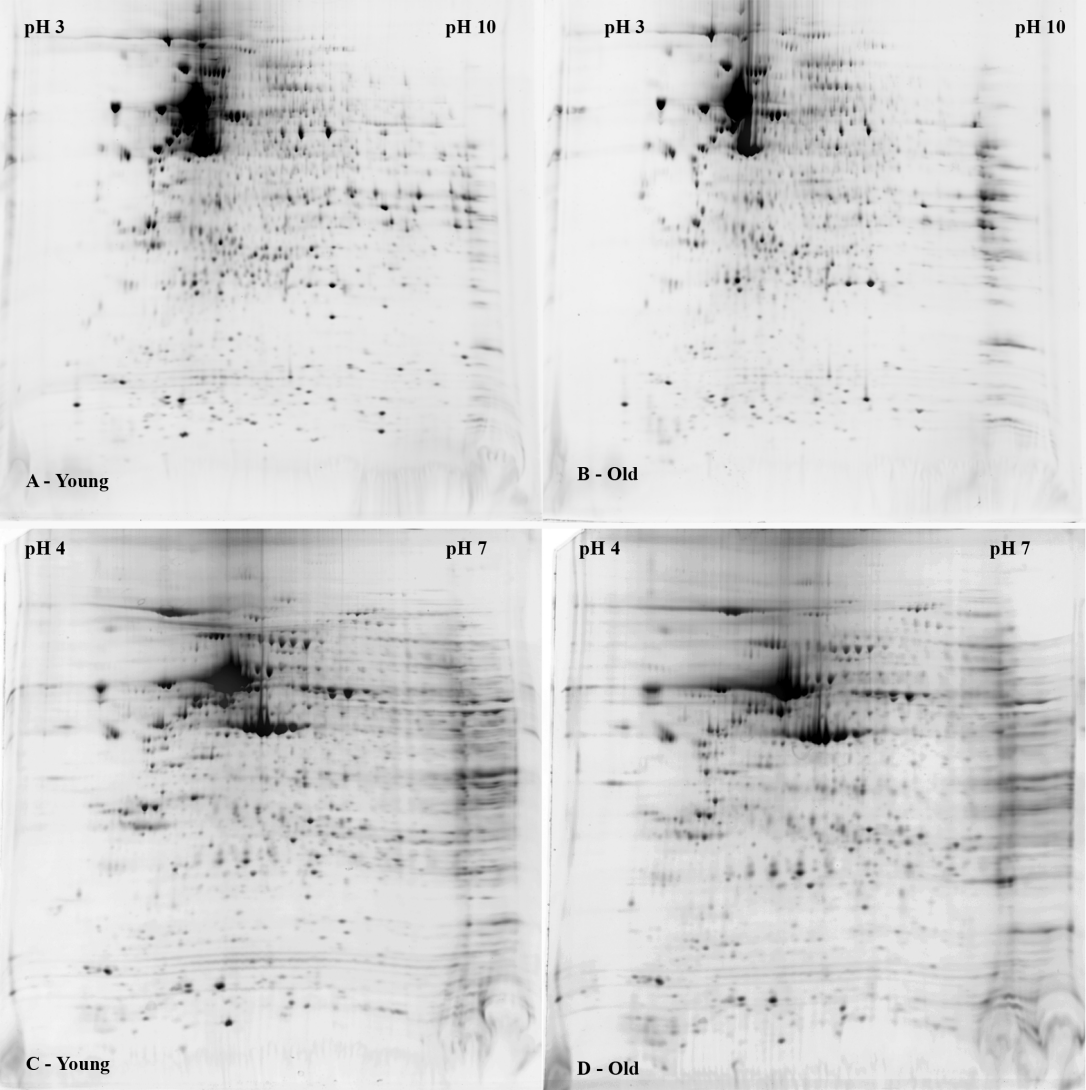
Representative two-dimensional gel images showing separation of proteins extracted from human corneal endothelial cells cultured from young and older donors. Extracted protein was pooled from five young (<30 years old; **A**,**C**) and five older donors (>50 years old; **B**,**D**). Equal amounts of protein were separated on either pH 3–10 IPG strips (**A**,**B**) or pH 4–7 IPG strips (**C**,**D**) followed by separation on 8%–16% polyacrylamide gels. Protein spots were stained with SyproRuby. Images were obtained using a ProEXPRESS Proteomic Imaging System.

Proteins were extracted from HCEC cultured from five individual donors per age group (five corneal pairs/group) and then pooled to form one sample per age group. We chose to analyze pooled samples to eliminate donor-to-donor variations in relative protein expression so as to detect specific age-related differences more easily. Besides the pooling of five individual samples, we also strictly followed a standardized 2-D separation protocol to eliminate gel-to-gel variation. Protein concentration of the two samples was determined just before the IEF first-dimensional separation. Equal amounts of protein were loaded for both gels. First- and second-dimensional separations of the two pooled samples were performed at the same time. Two-dimensional gel separations were repeated at least three times per sample, and the patterns were compared. Under these strictly controlled conditions, reproducible 2-D protein patterns were obtained using both pH 3–10 and pH 4–7 IPG strips. Figure 2A,B show typical 2-D patterns when samples were separated using pH 3–10 IPG strips. Overall, patterns from young donors (Figure 2A) were quite similar to those from older donors (Figure 2B), although several protein spots showed differences in relative density. Figure 2C,D show typical 2-D protein patterns when samples were separated using pH 4–7 IPG strips. The majority of protein spots observed using pH 3–10 IPG strips were also visible using pH 4–7 IPG strips. However, the pH 4–7 strip further separated proteins into more distinguishable spots with less streaking, thus achieving high-resolution spot separation to ensure accurate software analysis and spot-picking for protein identification. Within the maximum loading capacity of the IPG strip, more protein spots were revealed with increased protein loading, but the protein load needed to be balanced due to the tendency to induce streaking. Optimal resolution of protein spots for software analysis was obtained using a 400 μg protein load. SyproRuby was chosen for protein staining based on its excellent sensitivity and broad linear dynamic range [34].

### Differential analysis of two-dimensional gels from young and older donors

**Figure 3:**
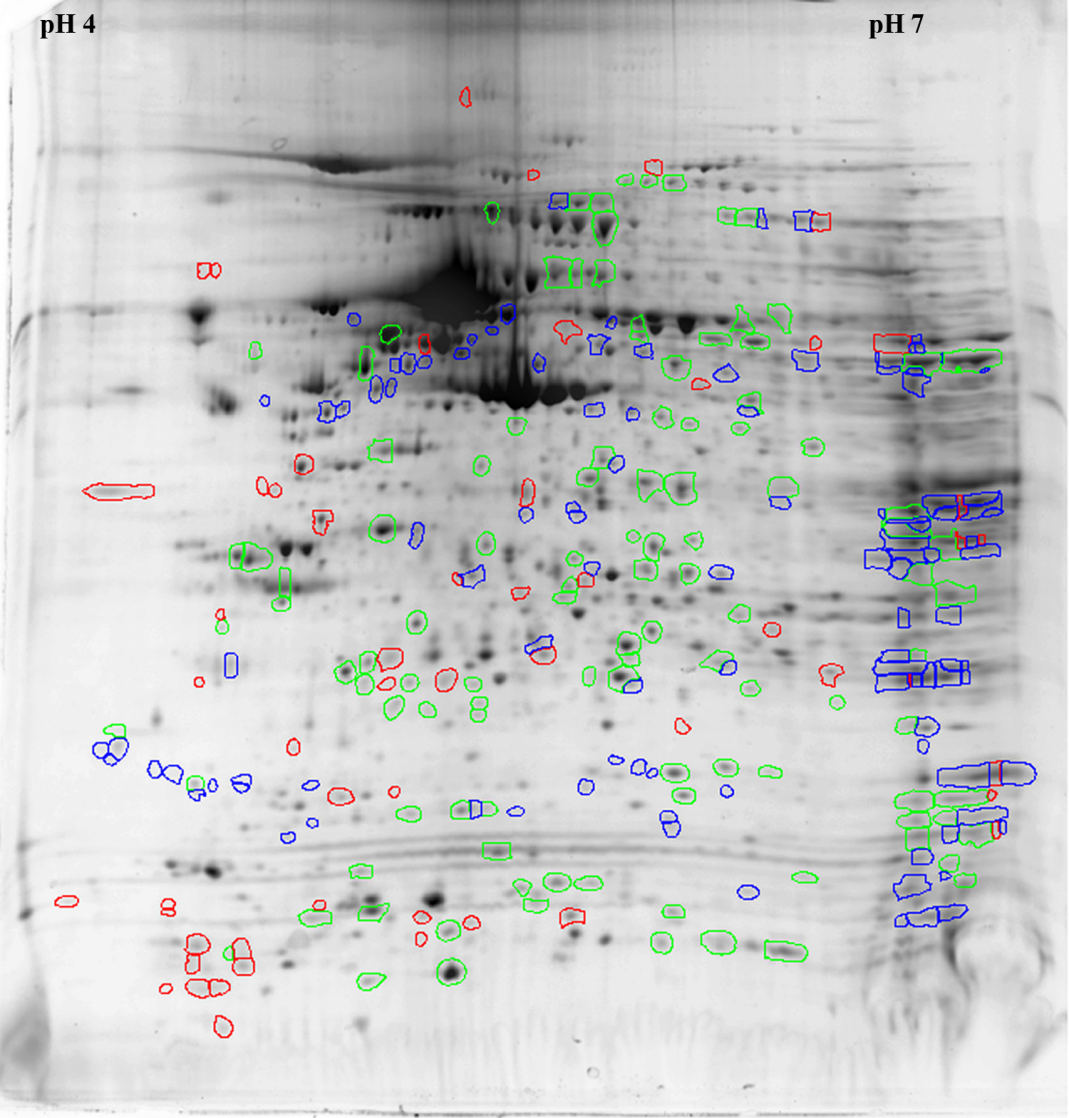
Relative differences in protein expression between human corneal endothelial cells cultured from young and older donors. The same 2-D gel from the young donor shown in Figure 2C has been enlarged and color-coded by the analysis software to indicate individual proteins spots that show differences in relative protein expression. Spots with no color designation indicate similar expression within a twofold range. Green indicates spots that were increased by twofold or greater in cells from young donors compared to older donors. Red indicates spots that were twofold decreased in young donors compared with older donors. Blue shows those spots that were present in extracts from young donors but were not detected in extracts from older donors.

The ProFinder software compared the normalized volume of individual spots between the two age groups. This form of analysis helped to eliminate any variations caused by protein loading or intra-strip (IPG) protein absorption differences and ensured that the comparison was conducted under well controlled conditions. The software automatically compared the spots based on identical anchoring spots present in both gels. The warping function corrected any distortion caused by changes in gel shape to achieve accurate spot matching. Figure 3 shows an enlarged image of the same 2-D gel from young donors as was presented in Figure 2C. However, in this figure, spots have been color-coded by the analysis software to indicate relative differences in protein spot density between HCEC from young and older donors. Spots not color-coded represent proteins in which comparative analysis indicated similar expression between the two age groups and in which differential analysis of normalized volumes indicated expression levels within a twofold range. The software also identified specific spots that were at least twofold different in normalized volume between the two age groups. In the figure, green circles indicate spots in which the normalized spot volume was increased at least twofold in young donors compared to older donors. Red circles indicate spots in which normalized volume was at least twofold decreased in young compared with older donors. Blue circles show those spots that were visible in 2-D gels from young donors but were unmatched in gels from older donors. Several spots in the gel from older donors were found by the software to be unmatched in the gel from young donors. The majority of those spots were located in the far right side of the gel, indicating that their isoelectric points were higher than pH 7. These spots are not indicated in the figure because they were insufficiently resolved under the isoelectric focusing conditions used for the current studies to make accurate quantitative comparisons or to identify by MALDI-TOF analysis.

### Protein identification

All detectable protein spots were picked and subjected to MALDI-TOF analysis and protein database identification. Appendix 1 presents an alphabetical listing of 58 proteins that showed similar expression levels (normalized volume) within a twofold range in HCEC from both young and older donors and that were identifiable by MALDI-TOF analysis. Multiple isoforms of some proteins are reported because their peptide profiles yielded different accession numbers upon search of the NCBI protein database. In a few cases, a single protein was resolved into multiple spots on the 2-D gels and yielded the same protein identification and database accession number upon MALDI-TOF analysis. In each of these cases, the position of the spot within the 2-D gel, the accuracy of the spot-picking, and accuracy of the protein identification were re-checked. Results suggest that these multiple spots represent single proteins that have undergone differential posttranslational modifications that alter the protein’s intrinsic charge and/or relative molecular weight. Appendix 2 presents a list of identified proteins in which the normalized volumes were at least twofold higher in HCEC from young donors compared to older donors, proteins that were expressed at least twofold higher in HCEC from older donors, and proteins that were identified in the gel from young donors that did not have a match in the gel from older donors. The list has been categorized into subgroups based on reported cellular function. Interestingly, several proteins known to function in cellular metabolism, antioxidant protection, protein folding or degradation, and cellular regulation were found to be expressed at least twofold higher in HCEC cultured from young donors.

## Discussion

Bioengineered corneal endothelium is being developed as a new form of treatment to restore visual acuity in patients with critically reduced ECD. Since such bioengineering requires culture of HCEC, its successful development requires exploration of the practicality of inducing proliferation in HCEC to increase cell numbers. In some cases, use of a patient’s own cells as a source for the bioengineered tissue would be optimal to avoid the risk of immune rejection. Because patients with low ECD are usually older, this treatment approach would necessarily involve the culture of cells that exhibit reduced proliferative capacity. Similarly, the majority of corneas available for use as a source of endothelial cells for bioengineered tissue will be obtained from older donors. For these reasons, it is important to explore age-related differences in the protein expression of cultured HCEC to identify differences in metabolism and other functions that may affect the ability of cells to divide and maintain a healthy state within the bioengineered construct. It is clear that donor age negatively affects the proliferative capacity of HCEC [12,14,18,19]. In cultured HCEC, this decrease in proliferative capacity is accompanied by changes in morphology and cell density at confluence with cells from older donors exhibiting an increase in cell size and decrease in overall cell density [14,18,19]. Such age-related changes in morphology were clearly demonstrated in the phase-contrast images presented in Figure 1. In the current study, HCEC were grown to passage 3 to increase total cell numbers so that sufficient protein would be available for proteomic analysis. The morphology and growth characteristics of the cells used in this study were very similar to those observed in previous studies in which HCEC were cultured from passage 1 to passage 4 [14,19,35], strongly suggesting that the cells used in the current study exhibited typical age-related characteristics and would therefore be representative of any cultured HCEC that could be used for the preparation of bioengineered tissue.

Proteomics technology provides a powerful high throughput screening tool to identify protein expression differences that may underlie age-related changes at the functional level. Protocols similar to those used in the current study have been used to conduct proteomic analyses of other ocular cells [25,31,36,37]. The experimental approach used for these studies involved collection of protein extracted from five individual donors within each age group. Equal amounts of protein from each of the five donors were then pooled to produce a single sample from each group. Sample pooling has been used by several researchers to avoid individual variations contributed by single samples [38-41]. Using pooled samples ensures that detected differences in relative protein expression better reflect common expression patterns within individual experimental groups. Repeated 2-D analysis of the two pooled samples of HCEC yielded patterns that were internally consistent. Comparison of the spot patterns and MALDI-TOF-based identification indicated a similar expression of at least 58 proteins within a twofold range. The decision to adopt the criterion of twofold differences was based on the recommendation of a specialist from Nonlinear Dynamics, the company that developed the proteomics analysis software used in this study. Differences in protein expression of twofold or greater have been used in the analysis of proteomic data by others [42-44].

Review of the data obtained in this study provides insight into changes in relative protein expression that suggest an age-related reduction in the ability of cultured HCEC to protect against oxidative stress and to maintain general cellular health. Of particular interest were changes indicating a potential age-related reduction in a subset of proteins involved in protecting against oxidative stress. Redox regulation is required to protect cells from oxidative stress, which is known to contribute to the aging process [45,46]. Proteomic studies [22] have identified several proteins involved in redox regulation in corneal tissue including glutathione S- transferase P, thioredoxin, and peroxiredoxins 1, 2, 5, and 6. In the current study, we found that glutathione synthetase, glutathione S-transferase, glutathione S-transferase omega, glutathione transferase P1–1, and peroxiredoxin-2 isoform-a were all expressed at least twofold higher in HCEC cultured from young donors, suggesting that HCEC cultured from older donors have decreased protection against oxidative stress compared to their younger counterparts. The finding that HCEC cultured from older donors express lower amounts of certain proteins involved in redox regulation correlates well with preliminary findings that HCEC both in ex vivo corneas and in culture exhibit an age-related increase in oxidative DNA damage [47] and that increased exposure of cultured HCEC to oxidative stress decreases relative proliferative capacity [48].

A comparison with the available gene expression profiles from human donor corneal endothelium [49-51] indicates a partial overlap with proteins identified in the current study. There are several reasons why only a partial overlap was obtained. One reason is that the gene expression studies were conducted using HCEC directly extracted from the cornea rather than from cultured cells. Posttranslational regulation of protein levels may also be responsible for some differences. In addition, results from the current study were based on a 400 μg protein load per gel. The protein spots visible with the SyproRuby staining most likely represented the majority of abundant proteins but may not have revealed proteins expressed at low levels. In addition, not all the protein spots detected by the software could be identified by matrix-assisted laser desorption/ionization time of flight (MALDI-TOF) mass spectrometry because there is a minimum protein requirement for accurate database identification of the digested peptides. This study was also restricted to those protein spots that separated within the pH 4–7 range. Thus, proteins with isoelectric points outside of this range would not be included. In addition, proteins with relative molecular weights greater than 100–120 kDa do not easily enter the IPG strip during the hydration step, thereby limiting the total number of proteins that can be separated by 2-D electrophoresis and identified.

The current studies are an initial effort to use proteomics technology to document age-related differences in the relative protein expression of cultured HCEC and are a first attempt toward understanding the molecular basis for age-related differences that may affect their relative proliferative capacity. Although the results obtained were internally consistent and appeared to reflect age-related differences observed in other cells, it is clear that the study has limitations in that additional, independent studies are needed to confirm the findings from this initial analysis before their relevance can be fully appreciated. Further study is also needed to determine whether the differences in relative protein expression identified in cultured HCEC are also present in HCEC in vivo. Identification of age-related differences in cultured HCEC should aid in the development of methods to optimally culture these cells for use in tissue bioengineering.

## Appendix 1. Proteins with similar expression between young and older donors.

To access the data, click or select the words “Appendix 1”. This will initiate the download of a (pdf) archive that contains the file. The asterisk denotes that the initial W/P stands for theoretical molecular weight/isoelectric point. The double asterisk indicates that the numbers in the column are National Center for Biotechnology Information accession numbers. Three asterisks indicates that the data in the column are the probabilities of correct protein identification based on Bayesian statistical analysis of protein sequences in the NCBI database.

## Appendix 2. Proteins with similar expression between young and older donors.

To access the data, click or select the words “Appendix 2”. This will initiate the download of a (pdf) archive that contains the file. The asterisk denotes that the initial W/P stands for theoretical molecular weight/isoelectric point. The double asterisk indicates that the numbers in the column are National Center for Biotechnology Information accession numbers. Three asterisks indicates that the data in the column are the probabilities of correct protein identification based on Bayesian statistical analysis of protein sequences in the NCBI database.
